# Reducing mortality in HIV-infected infants and achieving the 90–90–90 target through innovative diagnosis approaches

**DOI:** 10.7448/IAS.18.7.20299

**Published:** 2015-12-02

**Authors:** Shaffiq Essajee, Lara Vojnov, Martina Penazzato, Ilesh Jani, George K Siberry, Susan A Fiscus, Jessica Markby

**Affiliations:** 1 World Health Organization, Geneva, Switzerland; 2 Clinton Health Access Initiative, Boston, MA, USA; 3 Instituto Nacional de Saude, Maputo, Mozambique; 4 National Institutes of Health, Bethesda, MD, USA; 5 Department of Microbiology & Immunology, School of Medicine, University of North Carolina, Chapel Hill, NC, USA

**Keywords:** infant, HIV, diagnosis, treatment, point of care, SMS

## Abstract

**Introduction:**

Despite significant gains in access to early infant diagnosis (EID) over the past decade, most HIV-exposed infants still do not get tested for HIV in the first two months of life. For those who are tested, the long turnaround time between when the sample is drawn and when the results are returned leads to a high rate of loss to follow-up, which in turn means that few infected infants start antiretroviral treatment. Consequently, there continues to be high mortality from perinatally acquired HIV, and the ambitious goals of 90% of infected children identified, 90% of identified children treated and 90% of treated children with sustained virologic suppression by 2020 seem far beyond our reach. The objective of this commentary is to review recent advances in the field of HIV diagnosis in infants and describe how these advances may overcome long-standing barriers to access to testing and treatment.

**Discussion:**

Several innovative approaches to EID have recently been described. These include point-of-care testing, use of SMS printers to connect the central laboratory and the health facility through a mobile phone network, expanding paediatric testing to other entry points where children access the health system and testing HIV-exposed infants at birth as a rapid way to identify *in utero* infection. Each of these interventions is discussed here, together with the opportunities and challenges associated with scale-up. Point-of-care testing has the potential to provide immediate results but is less cost-effective in settings where test volumes are low. Virological testing at birth has been piloted in some countries to identify those infants who need urgent treatment, but a negative test at birth does not obviate the need for additional testing at six weeks. Routine testing of infants in child health settings is a useful strategy to identify exposed and infected children whose mothers were not enrolled in programmes for the prevention of mother-to-child transmission. Facility-based SMS printers speed up the return of laboratory results and may be of value for other testing services apart from HIV infant diagnosis.

**Conclusions:**

New tools and strategies for HIV infant diagnosis could have a significant positive impact on the identification and retention of HIV-infected infants. In order to be most effective, national programmes should carefully consider which ideas to implement and how best to integrate novel strategies into existing systems. There is no single solution that will work everywhere. Rather, a number of approaches need to be considered and should be linked in order to achieve the greatest impact on the continuum of care from testing to treatment.

## Introduction

The ambitious new global targets to identify 90% of all people living with HIV, treat 90% of those identified as infected and achieve virologic suppression in 90% of treated individuals by 2020 [1] represent a bold and timely call to identify new infections, promote sustained efforts to retain patients in care and reduce the morbidity and mortality associated with HIV infection. These so-called 90–90–90 targets have also been proposed for children in order to improve overall treatment coverage, which at 32% still lags behind the 40% for adults [2], and to reduce the high, early mortality of paediatric HIV, which peaks at age three to four months [3,4] and approaches 50% by two years of age [5].

Scale-up of services for early infant diagnosis (EID) is an essential step to address this problem, and some countries – especially those in Eastern and Southern Africa – have achieved remarkable success in this regard. In South Africa, for example, the decade between 2002 and 2012 saw a 100-fold increase in the number of EID tests performed. Data from the National Health Service Laboratory showed that in 2012 almost 75% of all HIV-exposed infants received their first EID test by two months of age [6]. The global statistics, however, are less encouraging. In the 22 Global Plan priority countries, only 50% of exposed infants received an EID test in the first two months of life during 2014 [7]. Moreover, it is estimated that of the infants identified as infected less than 35% are then referred for care and started on treatment [8], far from the goal of 90% tested and 90% treated.

There are inherent constraints associated with the current EID testing technologies that contribute to these poor performance statistics. The commercial platforms in use today require high-level facilities and highly trained technicians, which means that they can only be employed in well-resourced laboratories. Typically, a handful of such laboratories in each country serve many hundreds of service delivery sites, which are linked by specimen transport systems. Even in the best of circumstances, it can take four weeks for results to be returned to sites and in some settings it can take two months or more [9]. The purpose of this commentary is to review recent advances in EID that may help to overcome current constraints and to describe how these innovations may address some of the long-standing issues that have hampered access to testing and treatment.

## Discussion

**Figure 1:**
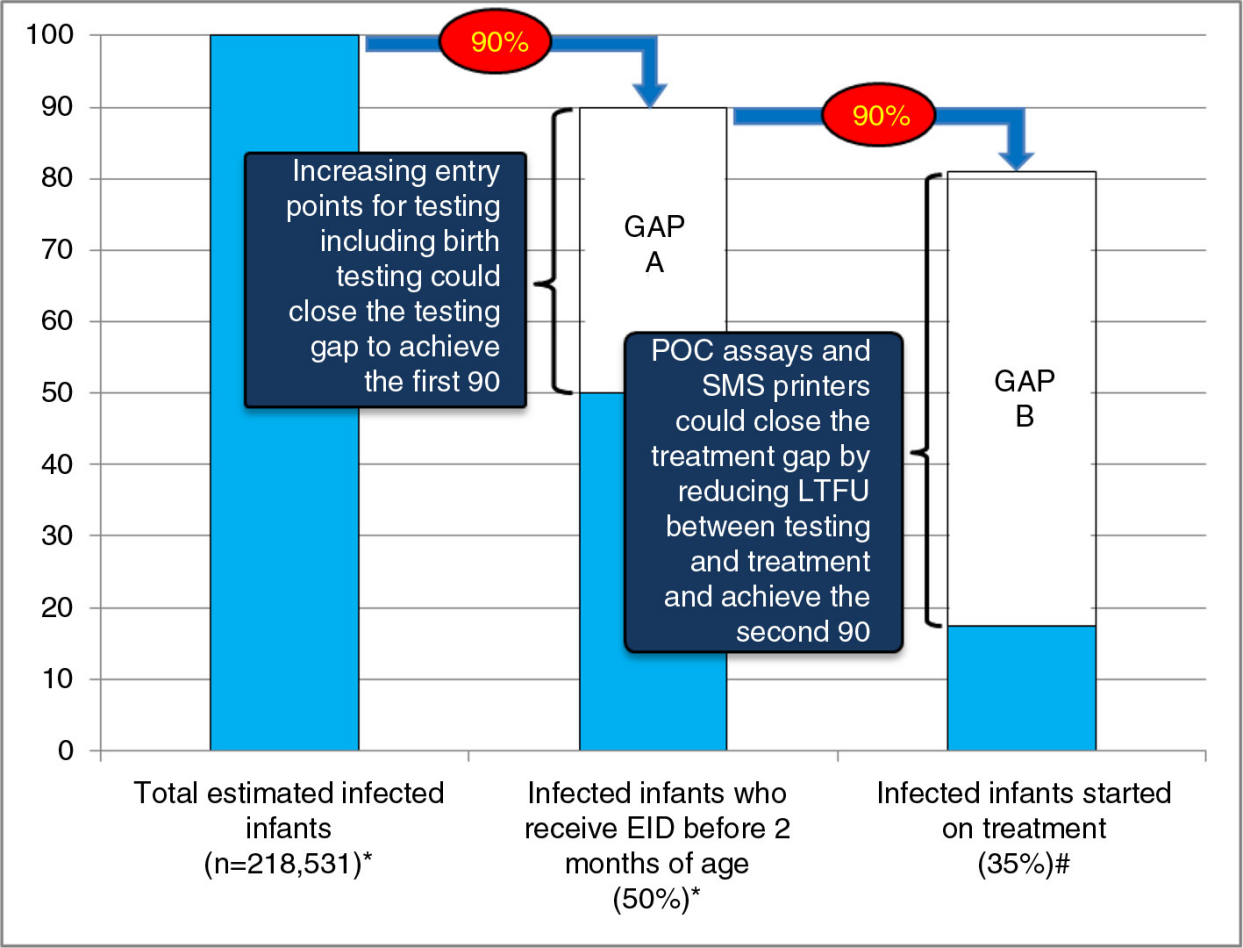
Estimated annual proportion of infected infants tested by two months of age in 2014 and estimated proportion of identified infected infants treated. Gap A represents the difference between infants currently receiving early infant diagnosis and the testing target of 90% of all positives, while Gap B represents the difference between infants treated and the treatment target of 90% of identified positives. Data sources: *Estimated number of new child infections in 2014 and estimated coverage of early infant diagnosis testing in 2014 taken from Global AIDS Response Progress Report, WHO, Geneva 2015 [7]. ^#^Proportion of infected infants receiving treatment from Chatterjee et al. *BMC Public Health*, 2011 [8].

Four innovations have recently emerged that may speed up identification and improve retention of infected infants to get us closer to 90% tested and 90% treated. Point-of-care (POC) EID tests and the use of SMS printers for result delivery are both examples of innovative tools that can decrease the turnaround time for results, potentially decreasing the time to initiation of antiretroviral therapy (ART) and reducing loss to follow-up. Expanding entry points for infant diagnosis beyond prevention of mother-to-child transmission (PMTCT) programmes could help to identify previously unrecognized HIV-exposed infants and “recapture” infants that have been lost to follow-up from the PMTCT continuum. In addition, virological testing at birth could detect *in utero* infected infants early and, with good linkage to care and prompt ART initiation, prevent mortality in such high-risk newborns. Figure 1 illustrates the testing and treatment cascade for HIV-infected infants and shows how these four innovative approaches could help to close the gap to achieve the 90% targets.

### Point of care EID

Rapid HIV antibody assays have been used successfully to decentralize HIV testing and facilitate widespread access to ART for adults. The key to this success is ease of use by non-laboratory personnel, thermostability of test supplies, low cost, high sensitivity and results within minutes at the point of care. In children under 18 months of age, however, antibody testing cannot be used for a definitive diagnosis due to the presence of maternal antibodies. To address this issue, a number of molecular technologies are in the pipeline for POC infant diagnosis and recently two of these, the Alere™ q HIV-1/2 Detect (Alere q) and the Cepheid Xpert^®^ HIV-1 (Xpert) assays, were approved by European regulators as *in vitro* diagnostic assays (CE-IVD) [10,11]. Both are polymerase chain reaction (PCR)-based technologies that provide results within 50 minutes (Alere q) to 90 minutes (Xpert) using “closed system” proprietary cartridges that contain all the constituent components for the PCR. Both have the potential of being able to quantify HIV viral load (VL), although the currently available version of Alere q does not have VL capabilities. The Xpert instrument can also be used to detect *Mycobacterium tuberculosis* (MTb) and identify rifampin resistance in MTb.

The Alere q runs one specimen at a time and can be used at primary health care facilities. It may also offer potential for mobile testing due to its portability (7.8 kg), ease of use and flexible power source options, including on-board battery. Alere q also requires small blood volumes (25 µL) and the test cartridge can be loaded from an infant heel-prick. The Xpert platform is more suited to district and regional hospitals due to the requirement for a constant power supply; however, this assay can be used with whole blood as well as dried blood spots (DBS). Although DBS use requires trained staff and additional laboratory equipment such as a thermomixer, it does make it possible to use the Xpert platform in a laboratory that is receiving DBS samples from several sites. Unlike Alere q, the Xpert device is modular and available in different sizes, which offers a range of throughput options for simultaneous specimen testing. The most commonly used four-module version can process 16 tests a day (assuming four specimens at a time and four test runs per eight-hour shift). The Xpert platform has been in use for several years for tuberculosis (TB) testing, and field experience has confirmed its suitability for higher level facilities [12]. Importantly, both instruments have built-in wireless connectivity, which can be used to track instrument utilization, supply chain, quality assurance and operator performance.

In comparative analyses, both assays perform well against the gold-standard laboratory-based PCR. Independent field evaluations of a prototype of the Alere q machine in Mozambique [13] and South Africa [14] showed sensitivity/specificity of 98.5/99.9% and 94.1/100%, respectively. Manufacturer-led laboratory evaluations of a prototype of the Xpert test using whole blood and DBS showed sensitivity of 95.6 to 98.2% and specificity of 98 to 98.5%, respectively. Independent field evaluations using the CE-IVD marked versions of the assays are currently underway and should be completed by the end of 2015. A small number of other POC technologies for EID are in the pipeline, with expected commercial availability in 2016.

POC EID testing offers great promise but comes with some limitations. The cost per test is higher than conventional laboratory-based PCR, although this may be offset in part by the fact that infants are more likely to actually get their results when using POC so the cost per test received may be comparable to laboratory-based PCR. Although test throughput may change as technologies evolve, appropriate placement of POC instruments should carefully consider current limits. There are very few EID collection sites that require more than 8 to 16 tests per day (the maximum throughput of the Alere q and Xpert platforms, respectively), so POC EID devices could be prioritized for placement at the largest sites in order to maximize the use of each device. It is also important to note that in the future many POC EID instruments will also have the ability to measure VL. Since the number of VL tests required will far outweigh the number of EID tests, placement of POC devices may ultimately be determined by the need for VL testing. To date, there is very little programme experience with implementing POC EID testing within health services. Practical considerations, such as how to ensure quality control, how to confirm a positive test result, when to start ART after a positive POC test and how to ensure that POC tests are captured within the national EID database, will all have to be addressed through targeted research and best practice learning from programmes. Overall, POC will likely not replace laboratory-based EID testing, especially in the short-medium term. Rather, POC EID will complement and enhance conventional approaches by offering a flexible and rapid testing approach that could be implemented by non-technical staff.

### SMS printers

Current EID testing programmes are typically built on a hub-and-spoke model of service delivery, where a small number of tertiary laboratories serve a large network of testing sites. In some countries, such as Uganda, one national laboratory provides centralized EID testing for the whole country [15]. Specimens are collected in the periphery and sent by courier or through the national post to central laboratories. Once results are available and confirmed, the laboratory sends back a paper copy of the results to the site using the same specimen transportation system. In theory this process should work well, but in practice delays are common and it is not unusual for results to take more than two months, by which time some infected infants may have died and others may be lost to follow-up. SMS technology enables results to be transmitted electronically to a small printer device at the site as soon as they are available in the laboratory. This technology has been implemented nationally in several countries including Kenya, South Africa, Mozambique, Zimbabwe, Rwanda and Zambia. A recent systematic review comparing turnaround time in paper-based versus SMS systems showed that SMS printers shorten the time until results are available by an average of 17 days [16]. For SMS printers to be deployed, there must be a cellular network through which data can be transmitted; although cellular services are now available almost everywhere, this approach may not work for sites that are especially remote. In addition, it is necessary to make an initial investment in printers at the site and computers in the laboratory, but in the long term the same system could be used to transmit any type of laboratory data.

### Expanded entry points for testing

For a number of reasons – either because HIV-positive women were never tested during antenatal care, or because they were lost from the PMTCT programme before delivery, or because they did not return with their infants for post-partum testing – 50% of HIV-exposed infants do not currently receive any EID testing in the first two months of life. However, because these infants may not have received the full complement of PMTCT interventions, they are also the ones at higher risk of acquiring HIV. In order to identify these children and bring them into care, it is important to expand infant and young child testing beyond EID in PMTCT programmes, by testing at other time points when children access the health system. This is an especially valuable strategy in high-prevalence settings but could also be of benefit in lower-prevalence settings in some circumstances. A recent systematic review found that in studies of testing of hospitalized children in Eastern/Southern Africa an average of 20.7% of all children tested were HIV-infected. In Western/Central Africa, the proportion was 10.8%. By contrast, testing in malnutrition clinics had a higher yield in Western/Central Africa compared with Eastern/Southern – 22 versus 6% [17]. The WHO has recommended provider-initiated HIV testing and counselling (PITC) in paediatric care settings for many years; however, few countries have implemented this guidance at the national level. The evidence suggests that using a universal testing approach for children admitted to hospitals or malnutrition centres would be a very successful approach for identifying HIV-infected and -exposed children and should be adopted widely. At present, EID is often offered as part of a specialized package of care that is seen as distinct and separate from routine infant and child health services. Integration of EID as a service provided at immunization clinics and well-child clinics may help to increase both uptake and follow-up, especially in countries with a high burden of HIV.

### Virological testing at birth

The high mortality of HIV in infants is driven in large part by the especially high risk of death among infants infected *in utero* [18]. Virological testing at birth has high specificity (99.7%) but low overall sensitivity (67.8%) due in large part to the fact that viral nucleic acids may not be detected at birth in infants who have acquired HIV through intra-partum transmission [19]. However, virological testing at birth testing should identify most infants who are infected through *in utero* transmission (although the precise impact of maternal antiretrovirals [ARVs] on the ability to detect *in utero* infection at birth is not known). If one of the goals of EID is to prevent infant mortality, then identifying and treating *in utero* infection as early as possible (with virological testing at birth linked to early treatment) may be a useful clinical strategy and one where POC EID could serve as a valuable tool to ensure that rapid action is taken to refer infants for ART once a newborn is confirmed positive. At the same time, it is important to recognize that for such an approach to be effective, a number of other elements need to be in place. Rates of facility delivery would have to be high enough to capture a significant proportion of infected newborns. Alternatively, for infants delivered at home, there would have to be a robust mechanism to find and test exposed babies in the first days of life, either through community outreach or by testing at BCG immunization, which is often administered three days after birth. Providers would need to be trained not only in testing neonates, but also in treating them. National procurement would have to be modified to buy more syrup ARV formulations, as the currently available Fixed Dose Combinations (FDCs) do not contain the correct dosing ratios for neonatal treatment. Moreover, we need additional research on what regimens to use in newborns, especially preterm and low birth weight infants, as there is a paucity of data on the dosing and safety of ARVs in this population. It will be critically important to develop and evaluate a testing approach that defines which infants might benefit most from a virological test at birth and how to manage a positive birth test. For infants that test negative at birth, close follow-up, tracking and repeat testing will be essential to retest at six weeks or soon after.

## Conclusions

Innovations in the tools available to perform EID may have a positive impact on the identification, retention and survival of HIV-infected infants and children. At the same time, the practical challenges of implementing strategies such as virological testing at birth and POC EID may hamper wide-scale adoption.

Where virological testing at birth and POC EID *are* implemented, they should be integrated with complementary strategies to enable infants to be followed across the continuum of care from testing through to treatment. For example, while POC testing will enable immediate identification of infected infants, having a test result in hand does not solve the problem of ensuring linkage to care for infected infants – especially if paediatric ARVs and staff trained in prescribing ART are not available. Elements of an integrated approach include the following: task shifting of paediatric testing to enable more providers to test children (including training a cadre of staff for POC testing); mentoring of clinicians and improved registers to identify early loss to follow-up of infants; robust “leak-proof” mechanisms to refer infected infants and children for treatment; training and equipping clinicians with the knowledge, tools and commodities they need to treat infected infants; and systems to continuously monitor the quality of testing as well as retention and impact on child survival.

In order to achieve the 90–90–90 goal for children, we cannot rely on any one-size-fits-all solution. Rather, we must develop context-specific, integrated, multifaceted approaches that involve identification and treatment of pregnant HIV-positive women as early as possible, prevention of loss to follow-up for mother–infant pairs, provision of timely paediatric diagnosis using technologies that are best suited to the setting where they are deployed and robust linkage of infected infants and children to lifelong care and treatment. It is important to note that not all improvements need novel technological solutions. In many cases, simple interventions, such as strengthening formal record-keeping and introducing systems to track mothers and infants who fail to return for follow-up visits, may increase retention within existing infrastructures. Finally, it is vital to recognize that community engagement to ensure acceptability and uptake of testing is a key enabler to achieve the greatest impact [20]. Without buy-in from communities of pregnant women and mothers living with HIV, even the most sophisticated of tools will not address the high rate of loss to follow-up and mortality and poor access to treatment for children.
